# Resistance of Lima Bean (*Phaseolus lunatus* L.) to the Red Spider Mite *Tetranychus neocaledonicus* (Acari: Tetranychidae)

**DOI:** 10.3389/fpls.2018.01466

**Published:** 2018-10-11

**Authors:** Solange Maria de França, Paulo Roberto Ramalho Silva, Antonio Vieira Gomes-Neto, Regina Lucia Ferreira Gomes, José Wagner da Silva Melo, Mariana Oliveira Breda

**Affiliations:** ^1^Agronomy-Tropical Agriculture Postgraduate Program, Agricultural Science Center-Entomology, University Campus Minister Petrônio Portella – Universidade Federal do Piauí, Teresina, Brazil; ^2^Agricultural Science Center-Entomology, University Campus Minister Petrônio – Universidade Federal do Piauí, Teresina, Brazil; ^3^Center of Agricultural Sciences, Department of Plant Science, Universidade Federal do Ceará, Fortaleza, Brazil; ^4^Laboratory of Agricultural and Forest Entomology, Center of Agricultural Sciences, Department of Agronomy, Universidade Federal de Alagoas, Rio Largo, Brazil

**Keywords:** antibiosis, antixenosis, population growth, host choice, *Phaseolus lunatus*, Tetranychidae

## Abstract

The red spider mite, *Tetranychus neocaledonicus* (Acari: Tetranychidae) can be an important pest on lima bean (*Phaseolus lunatus* L.). Thus, the objective of this work was to assess the antibiosis and antixenosis effects of lima bean genotypes on *T. neocaledonicus*, through the evaluation of performance parameters as well as the host preference for food and oviposition. Nine lima bean genotypes from the Active Bank of Germplasm of the Federal University of Piauí – BGP / UFPI were screened. To assess antibiosis parameters, eggs of *T. neocaledonicus* were individually placed on leaf disks of each genotype. The period and survival of the different stages of development (larvae, protonymph, deutonymph and adult), pre-oviposition, oviposition and post-oviposition period, longevity and fecundity of females were evaluated, and fertility life table parameters were calculated. In choice tests, adult females of *T. neocaledonicus* were used. The numbers of mites and eggs were counted for each genotype. The protonymph, egg-adult, longevity and oviposition period, fertility life table parameters, as well as the food and oviposition preference were affected by lima bean genotypes. We found that some genotypes reduced adult female longevity, increased the larval and egg-adult period, decreased oviposition period, negatively affected the fertility life table parameters, reducing the net reproductive rate (R_o_), the intrinsic rate of increase (r_m_) and the finite rate of increase (**λ**), while increasing the population doubling time (DT), exhibiting a reliable antibiosis effect upon *T. neocaledonicus*. Nevertheless, these same genotypes were the most preferred for food and oviposition. By contrast, some other genotypes reduced the adult female longevity and oviposition period, elongated the larval period and affected fertility life table parameters, demonstrating an antibiosis effect upon *T. neocaledonicus.* Moreover, these other genotypes were among the less preferred for food and oviposition, exhibiting an additional antixenosis effect. Thus, our results demonstrate that the genotypes of lima bean may present distinct levels of resistance to *T. neocaledonicus*, and this resistance may be an important tool for Integrated Pest Management. This is one of the first studies aiming to describe mite resistance sources in lima bean.

## Introduction

The use of host plant resistance is widely known as an efficient, economical, ecological and socially advantageous control method within Integrated Pest Management (IPM) programs (Stenberg, 2017). Plant resistance against herbivores is composed of two parts: antibiosis, affecting the pest’s fitness, resulting in reduced population growth, longevity and higher mortality; and antixenosis, the non-preference behavior of the pest for feeding, oviposition or shelter (Dehghan et al., 2009; Silva et al., 2011; Stenberg and Muola, 2017).

Some studies aiming to select host plant resistance to pest mites have been carried out in recent decades. Sources of resistance to *Tetranychus evansi* Baker & Pritchard (Acari: Tetranychidae) were investigated in tomato varieties (Silva et al., 1992); strawberry and vines sources were tested for *T. urticae* Koch (Acari: Tetranychidae) resistance (Lourenção et al., 2000; Valadão et al., 2012; Breda et al., 2016) evaluated sweet pepper genotypes’ resistance to the broad mite, *Polyphagotarsonemus latus* (Banks) (Acari: Tarsonemidae). Silva et al. (2011) evaluated Rubber tree clones’ resistance to Eriophyiidae and Tenuipalpidae mites, among other studies. Here, for the first time, we study the resistance of lima bean (Fabaceae) genotypes to pest mites.

Lima bean has a significant economic and social importance, with features of tolerance to drought and heat, which justify its economic exploitation, primarily in family farming, as one of the main sources of income and livelihood, contributing to food security (Baudoin, 1988; Vieira, 1992; Santos et al., 2002). Therefore, several studies aiming to increase the genetic knowledge about lima bean have been carried out, through the collection of traditional and wild genotypes, molecular characterization and maintenance of gene banks (EMBRAPA, 2016).

In addition, several species of Tetranychidae mites are reported in association with lima bean, including *T. urticae*, *Eutetranychus banksi*, and *T. neocaledonicus* (Mendonça et al., 2011; Silva and Gondim, 2016; Gomes Neto et al., 2017). Throughout the world, *T. neocaledonicus* can be found on more than 400 different host plants, presenting itself as a species of considerable economic importance for several crops, with a wide distribution in the intertropical zone (Gutierrez and Zon, 1973; Bonato and Gutierrez, 1999).

The present study aimed to evaluate host plant resistance aspects of nine genotypes of lima bean from the Active Bank of Germplasm of the Federal University of Piauí – BGP / UFPI to the red mite, *T. neocaledonicus*.

## Materials and Methods

### *Phaseolus lunatus* L. (Fabaceae) Genotypes

Nine lima bean genotypes from the Active Bank of Germplasm of the Federal University of Piaui – BAGF / UFPI were used. The genotypes and their features are included in **Table 1**. The genotypes were chosen based on widespread use by farmers and prior studies of agronomic and molecular features, as well as disease resistance developed in the region.

**Table 1:** Genotypes of lima bean from the Active Bank of Germplasm of the Universidade Federal do Piauí- BGP / UFPI used in the bioassays, and their features.

Genotype	Origin	Common name	Weight^∗^ (g)
UFPI-822	Puxinanã – PB	Coquinho bean	38.81
UFPI-883	Esperantina – PI	White bean	48.49
UFPI-887	Novo Oriente – CE	Mestiça bean	69
UFPI-888	Tianguá – CE	White bean	56.64
UFPI-891	Uruçuí – PI	White bean	56.64
UFPI-908	Pedra Branca – CE	Butter bean	49.62
UFPI-909	Crato – CE	White bean	57.84
UFPI-915	Miguel Alves – PI	White bean	57.91
UFPI-971	Barras – PI	Boca de Moça bean	64.57

*^∗^Weight of 100 g of seeds.*

### *Tetranychus neocaledonicus* André (Acari: Tetranychidae) Rearing

The mites were reared in the Phytotechnical Department of the Federal University of Piaui (UFPI), under greenhouse and laboratory conditions, on lima bean. Plants of the genotype UFPI-971-PI were grown in plastic containers of 3.8 liters in the greenhouse. Thirty days after emergence, the plants were infested with *T. neocaledonicus* females. Weekly, during the whole period of bioassays, new infestations were made through direct contact between plants infested with mites and uninfested plants. Under laboratory conditions, leaf disks of lima bean UFPI-971-PI were infested on filter paper moistened by a water-saturated sponge. The leaf disks were surrounded by hydrophilic cotton wool to prevent mite escape. The bioassays and *T. neocaledonicus* rearing were carried out with temperature and relative humidity monitored daily by a thermohygrograph, and 12 h photoperiod.

### Antibiosis of Lima Bean Genotypes

To assess the antibiosis effects of the nine genotypes of lima bean on *T. neocaledonicus*, the performance aspects of the red spider mite were evaluated. Leaf disks of 3 cm diameter, from 30-day-old lima bean plants, medium leaves, of each genotype, were kept on filter paper over a moistened sponge in Petri dish arenas. Three *T. neocaledonicus* adult females were left for an oviposition period of 16h in each disk. After this period, the females were removed, and one egg was kept per arena.

The following parameters were evaluated: incubation period (days) (period between egg oviposition and larval hatching), egg viability (%), period of development stage (larva, protonymph, deutonymph and adult) and egg-adult period. Two scorings were performed per day, every 12 h (7 a.m. and 7 p.m.) during larval stage. At the adult stage, the following parameters were daily evaluated: pre-oviposition (period prior to the first oviposition), oviposition and post-oviposition periods (days), female fecundity, fertility and longevity.

Every 7 days the leaf disks were replaced, and the mites transferred with the aid of a fine brush. The experiment was kept in a BOD incubator (Bio-Oxygen Demand) with a temperature of 25 ± 1° C, relative humidity of 70 ± 10% and photophase of 12 h. A completely randomized design was used with nine treatments (lima bean genotypes) and 20 replicates. The data were (*x* + 1)^0.5^ transformed to satisfy the ANOVA assumptions. The Scott and Knott grouping test was performed, using the SISVAR statistical program (Ferreira, 1998).

The cluster analysis was performed using the Unweighted Pair Group Method with Arithmetic Mean (UPGMA), which groups individuals (lima bean genotypes) according to similarity (antibiosis and antixenosis effects on *T. neocaledonicus*), considering the parameters of incubation period, period of development stages (larva, protonymph, deutonymph), egg-adult period and longevity. The analysis was performed by the PAST software (Hammer et al., 2001).

For fertility life table parameters, the survival rate (l_x_), specific fertility (m_x_), net reproductive rate (R_o_), intrinsic rate of increase (r_m_), mean generation time (T), finite rate of increase (λ) and population doubling time (DT) were calculated to provide accurate data for the determination of host quality, thus helping the identification of possible resistance sources (Gomes Neto et al., 2017). The data were submitted to Analysis of variance with the Duncan test at 5% probability, by the statistical program SAS (SAS Institute, 2001).

### Antixenosis of Lima Bean Genotypes

To assess the antixenosis effects of the nine genotypes of lima bean on *T. neocaledonicus*, food and oviposition choice tests were performed. Petri dishes of 15 cm diameter with filter paper over a moistened sponge were used as arenas. In the center of each arena, a plastic disk of 8 cm diameter was placed and surrounded by leaf disks of 3 cm diameter of each genotype. The leaf disks were placed equidistant from the center of the arena in contact with the plastic disk. Twenty adult females of *T. neocaledonicus* were released in the central plastic disk. After 1, 3, 6, 12, 24, and 48 h, the number of mites and eggs were evaluated. The bioassay was developed at 25 ± 1°C, 70 ± 10% RH and 12 h of photophase. The analysis was performed by the Scott and Knott (1974) grouping test at 5%, using the statistical program ASSISTAT.

## Results

### Antibiosis of Lima Bean Genotypes

**FIGURE 1:**
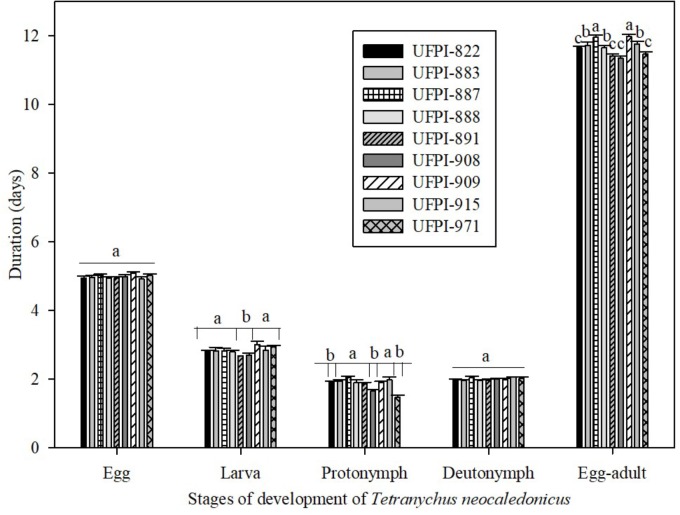
Effects lima bean genotypes on the development period (days; Means ± SE) of *Tetranychus neocaledonicus* females. 25 ± 1°C, RH 70 ± 10% and 12 h photophase. Means followed by the same letters in stage of development do not differ statistically by the Scott-Knott at 5% probability.

Regarding the antibiosis effect of lima bean on the development period of *T. neocaledonicus*, the nine tested genotypes did not affect the incubation (eggs) and the deutonymph period of the mite, with mean values of 4.98 and 1.81 days, respectively. However, the lima bean genotypes significantly affected the larval, protonymph and egg-adult periods (**Figure 1**).

Genotypes UFPI-891 and UFPI-908 significantly reduced the larval period of *T. neocaledonicus*, with means of 2.68 and 2.70 days. The highest value for larval period was observed on the UFPI-909 genotype (3.0 days), not differing from the other genotypes. Genotypes UFPI-908, UFPI-822 and UFPI-971 significantly reduced the protonymph period (1.65, 1.57 and 1.47 days). The longest egg-adult periods were observed on genotypes UFPI-909 and UFPI-887 (11.98 and 11.96 days), significantly differing from the other genotypes, while the shortest egg-adult period occurred for UFPI-908 (11.35 days), although not differing from UFPI-882, UFPI-891, UFPI-908, and UFPI-971.

None of the lima bean genotypes affected egg-larval, protonymph, deutonymph or egg-adult survival rates. However, for genotypes UFPI-888, UFPI-908, and UFPI-915 a higher female longevity was observed, with mean values of 47.90, 47.55, and 47.25 days, respectively. However, UFPI-822 caused the lowest adult female longevity (39.47 days), although not differing from UFPI-883, UFPI-887, UFPI-891, UFPI-909, and UFPI-971 (**Table 2**).

**Table 2:** Effects of lima bean genotypes on the survival rate (%) and adult female longevity (days) of *Tetranychus neocaledonicus*. 25 ± 1°C, RH 70 ± 10% UR and 12 h photophase.

Genotypes	Survival rate (%)				Longevity
	Egg-larvae	Protonymph	Deutonymph	Egg-adult	
UFPI-822	95	94.74	88.89	80	39.47 ± 1.50 b
UFPI-883	100	95	89.47	85	41.30 ± 0.49 b
UFPI-887	100	95	89.47	85	43.25 ± 0.78 b
UFPI-888	100	95	94.44	85	47.90 ± 0.21 a
UFPI-891	96	95	94.74	90	41.02 ± 0.45 b
UFPI-908	100	100	100.00	95	47.55 ± 0.88 a
UFPI-909	100	95	89.47	85	43.55 ± 1.66 b
UFPI-915	100	95	94.74	90	47.25 ± 0.72 a
UFPI-971	100	95	94.74	90	44.32 ± 2.38b

*^∗^Means followed by the same letters in the columns do not differ statistically by Scott–Knott at 5% probability.*

Regarding the effect of lima bean upon *T. neocaledonicus* oviposition periods, genotypes UFPI-915, UFPI-888, UFPI-908, and UFPI-971 significantly lengthened the oviposition period, with values of 45.22, 45.19, 45.02 and 42.02 days, respectively. Among the genotypes, UFPI-822 significantly promoted the lowest oviposition period (37.02 days). Nevertheless, the lima bean genotypes did not affect pre and post-oviposition periods (**Table 3**).

**Table 3:** Effects of lima bean genotypes on the preovipostion, oviposition and post-oviposition periods (days; means ± SE) of *T. neocaledonicus*. 25 ± 1°C, RH 70 ± 10% and 12 h photophase.

Genotypes	N^1^	Pre-oviposition ± SE	Oviposition ± SE	Post-oviposition ± SE^2^
UFPI-822	10	1.21 ± 0.12a	37.02 ± 0.60c	1.30 ± 0.42a
UFPI-883	11	1.10 ± 0.14a	39.02 ± 0.40b	1.20 ± 0.23a
UFPI-887	11	1.41 ± 0.10a	40.12 ± 0.30b	1.31 ± 0.33a
UFPI-888	12	1.51 ± 0.18a	45.19 ± 0.33a	1.29 ± 042a
UFPI-891	15	1.11 ± 0.04a	39.02 ± 0.44b	1.23 ± 0.19a
UFPI-908	13	1.23 ± 0.09a	45.02 ± 0.60a	1.22 ± 0.20a
UFPI-909	11	1.39 ± 0.14a	41.02 ± 0.60b	1.27 ± 0.42a
UFPI-915	14	1.36 ± 0.13a	45.22 ± 0.49a	127 ± 0.19a
UFPI-971	12	1.81 ± 0.18a	42.02 ± 0.47a	1.28 ± 0.42a

*^1^Number of observations; ^2^Means followed by the same letters in the columns do not differ statistically by Scott–Knott at 5% probability.*

**FIGURE 2:**
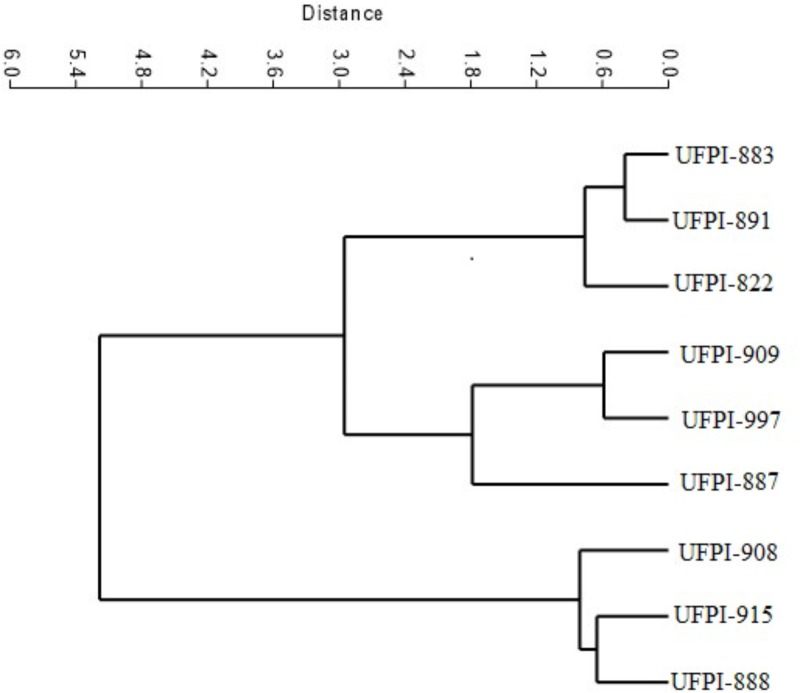
Phenogram representative of the effect of lima bean genotypes on the biology of *T. neocaledonicus*.

The cluster analysis allowed the formation of three distinct groups of lima bean genotypes: the first group with genotypes UFPI-908, UFPI-915, and UFPI-888 (Group 1), the second group with genotypes UFPI-909, UFPI-887, and UFPI-971 (Group 2) and the third with genotypes UFPI-883, UFPI-891, and UFPI-822 (Group 3) (**Figure 2**). Among the tested parameters for *T. neocaledonicus*, the greatest influence on cluster analysis was adult female longevity. In the first group are the lima bean genotypes which provided the highest female longevity. In the second group, there are genotypes with intermediate values for female longevity and, in the third group, the genotypes with the lowest female longevity values.

Lima bean genotypes significantly affected the fertility life table parameters of *T. neocaledonicus* (**Table 4**). For the net reproductive rate (R_o_) genotypes UFPI-887 and UFPI-909 presented the lowest values (21.08 and 20.18), significantly differing from the other genotypes. The mean generation time (T) ranged from 15.44 to 21.23 days for UFPI 888 and UFPI-971, with genotypes UFPI-882, UFPI-891, UFPI-908, UFPI-915, and UFPI-971 presenting significantly higher values. The intrinsic rate of increase (r_m_) was significantly reduced by genotypes UFPI-909 and UFPI-887, with values of 0.13 and 0.14 individuals/female/day, and the population doubling time (DT) was significantly increased by genotypes UFPI-887 and UFPI-909, with values of 5.33 and 5.32 days, respectively.

**Table 4:** Fertility life table parameters of *T. neocaledonicus* (means ± SE) on lima bean genotypes. 25 ± 1 °C, RH 70 ± 10% and 12h photophase.

P^1^	UFPI-822	UFPI-883	UFPI-887	UFPI-888	UFPI-891	UFPI-908	UFPI-909	UFPI-915	UFPI-971
r_m_	0.27 ± 0.03a	0.19 ± 0.013b	0.14 ± 0.01c	0.18 ± 0.02b	0.26 ± 0.03a	0.25 ± 0.03a	0.13 ± 0.06c	0.20 ± 0.032b	0.25 ± 0.02a
T	19.24 ± 0.17a	17.12 ± 0.009b	18.32 ± 0.17b	15.44 ± 0.11c	20.21 ± 0.21a	21.09 ± 0.09a	18.29 ± 0.33b	20.18 ± 0.19a	21.23 ± 0.31a
R_0_	29.13 ± 0.67a	27.25 ± 0.53a	21.08 ± 0.13c	25.19 ± 0.29b	27.13 ± 0.37a	27.27 ± 0.49a	20.18 ± 0.36c	25.33 ± 0.27b	26.17 ± 0.67a
λ	1.30 ± 0.01a	1.20 ± 0.01b	1.12 ± 0.02c	1.19 ± 0.01b	1.22 ± 0.32b	1.28 ± 0.03a	1.14 ± 0.02c	1.21 ± 0.037b	1.29 ± 0.018a
DT	4.58 ± 0.06b	4.59 ± 0.18b	5.33 ± 0.10a	3.43 ± 0.012c	4.14 ± 0.017b	3.42 ± 0.014c	5.32 ± 0.12a	4.65 ± 0.13b	4.69 ± 0.06b

*^1^ P: Fertility life table parameters; r_m_: Intrinsic rate of increase; T: Mean generation time; R_0_: Net reproductive rate; λ: Finite rate of increase and DT: Population doubling time. Means followed by the same letters in the rows do not differ statistically by the Scott–Knott test, at 5% probability*

### Antixenosis of Lima Bean Genotypes

**FIGURE 3:**
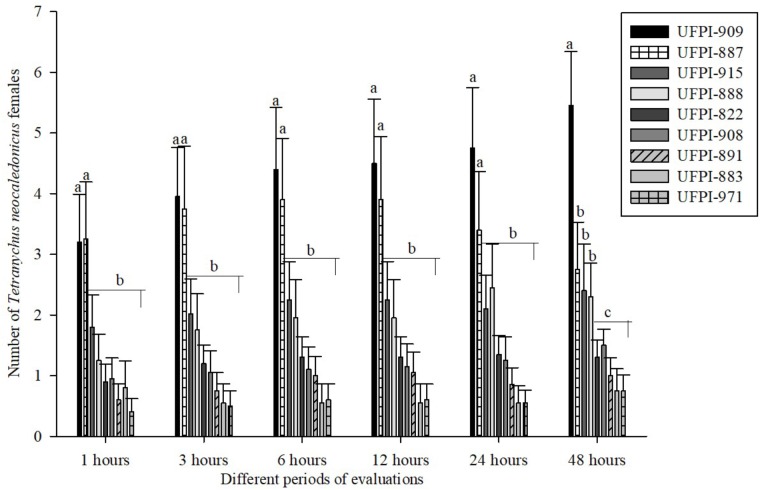
Food preference of *T. neocaledonicus* adult female (Means ± SE) on lima bean genotypes after 1, 3, 6, 12, 24, and 48 h. Means followed by the same letters in stage in each time interval do not differ statistically by the Scott-Knott at 5% probability.

Regarding the antixenosis effect of lima bean genotypes on *T. neocaledonicus*, the choice tests for food preference demonstrated that host selection started 1 h after exposure to the lima bean genotypes. Genotypes UFPI-909 and UFPI-887 were the ones most chosen among the nine tested genotypes after 24 h of bioassay. After 48 h, UFPI-822, UFPI-908, UFPI-891, UFPI-883, and UFPI-971 were the least chosen among the nine lima bean genotypes (**Figure 3**).

**FIGURE 4:**
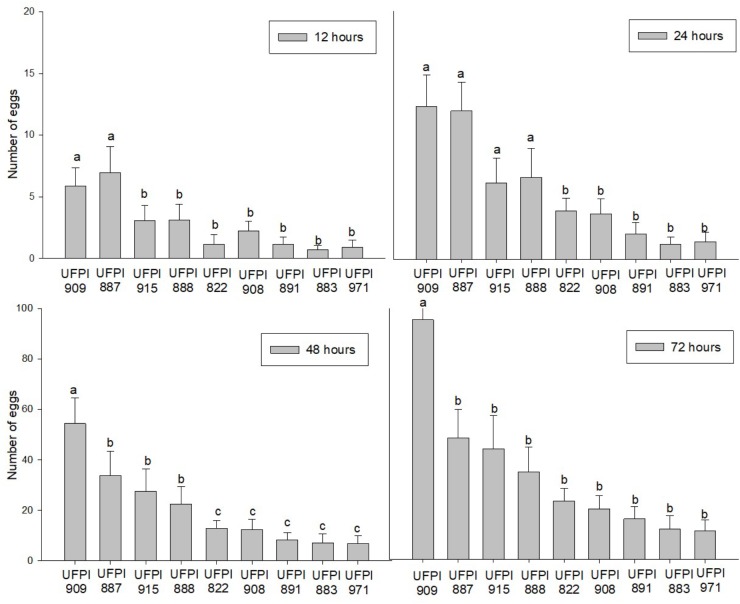
Number of eggs (Means ± SE) of *T. neocaledonicus* on lima bean genotypes after 12, 24, 48, and 72 h. Columns with the same letter at each evaluation interval do not present statistically differences by the Scott-Knott at 5% probability.

The number of eggs was directly proportional to the time of exposure for the nine tested lima bean genotypes. However, UFPI-909 and UFPI 887 presented the highest number of eggs after 24 h, among the tested genotypes. On the other hand, UFPI-822, UFPI-908, UFPI-891, UFPI-883, and UFPI-971 presented the lowest number of eggs during the whole evaluation period, indicating a possible antixenosis effect for oviposition (**Figure 4**).

## Discussion

Overall, genotypes UFPI-887 and UFPI-909, group 2 in the cluster analysis, reduced adult female longevity, increased the larval and egg-adult period, decreased oviposition period and negatively affected the fertility life table parameters, reducing the net reproductive rate (R_o_), the intrinsic rate of increase (r_m_) and the finite rate of increase (**λ),** while increasing the population doubling time (DT). Fertility life table parameters have been used successfully to determine host-plant quality and to identify sources of antibiosis resistance (Razmjou et al., 2009; Lin, 2013). For that, it is possible to determine that genotypes UFPI-887 and UFPI-909 exhibited a reliable antibiosis effect upon *T. neocaledonicus*. Nevertheless, these same genotypes were the most preferred for food and oviposition. According to Valladares and Lawton (1991), a poor link between host plant choice by the adult insect/mite and offspring performance has been widely observed and may be explained by several hypotheses, as host preference is often based on many factors, such as competition, microclimate, host density, size, age and chemical features, among others.

Based on the findings of the present study, genotypes UFPI-887 and UFPI-909 could be suggested as promising plants for trap cropping development strategies in IPM programs for *T. neocaledonicus*. Trap cropping is based on distinct herbivore preference among plant species, genotypes or crop stages (Hokkanen, 1991). The strategy’s development may consist of offering the preferred genotype in space and time of the main crop, manipulating the pest mite population. Crop protection could be achieved by preventing the mite population from colonizing the main crop or by trapping them on a genotype with strong antibiosis, which could be colonized without affecting the main crop. Javaid and Joshi (1995) also reported that trap cropping might involve early planting of border strips of a genotype to attract the pest mites to a place where they may well be exposed to chemical control. For that, additional studies should be performed to confirm the genotype preference and antibiosis effect upon *T. neocaledonicus* at field scales.

By contrast, genotypes UFPI-882, UFPI-891, and UFPI-883, group 3 in the cluster analysis, reduced the adult female longevity and oviposition period, elongated the larval period and affected fertility life table parameters, demonstrating an antibiosis effect upon *T. neocaledonicus.* Moreover, these genotypes were among the less preferred for food and oviposition, exhibiting an additional antixenosis effect.

Combining strong antibiosis and antixenosis, the three genotypes UFPI-882, UFPI-891, and UFPI-883 could be used as sources of resistance to *T. neocaledonicus* in future programs. According to Sharma and Ortiz (2002), one of the major attractive features of plant resistance to herbivorous predators is that it does not require farmers to have any specific skill for the employment of the technique. Furthermore, the financial investment by farmers is very low. For the lima bean crop context of family farming in underdeveloped regions, the advance of resistant genotypes to a potential pest threat presents itself as a management practice that ensures food sovereignty.

Altogether, the nine lima bean genotypes increased the egg-adult development period of *T. neocaledonicus*, ranging from 11.35 to 11.98 days at 25°C, when compared to values found in the literature for other Tetranychidae mites on *Phaseolus* hosts. According to Rivero and Vásquez (2009), *T. desertorum* Banks on lima bean presented an egg-adult period of 6.8 days at 28°C. Morros and Aponte (1995) reported that *T. ludeni* Zacher also presented an egg-adult period of 6.8 days on *Phaseolus vulgaris* L. at 26°C.

According to Ballhorn et al. (2005), lima bean is already well described as emitting a toxic compound, cyanide, as well as volatile organic compounds (VOCs) for plant defense against herbivorous predators. Such defenses are assumed to protect the plant directly, reducing oviposition and/or feeding; and indirectly affecting the herbivore’s development. Furthermore, the effects of VOCs are varied and may even include repellence in herbivores (Moraes et al., 2001; Heil, 2004).

Although some evidence may infer that piercing-sucking herbivores do not promote enough tissue disruption to activate plant defense process (Schreiner et al., 1984), laboratory studies recognize that the release of VOCs may be induced by minor injuries from small piercing-sucking herbivores, for instance, spider mites (Dicke, 1999; Ballhorn et al., 2008).

Besides that, in the present study, the nine lima bean genotypes significantly affected the performance and population growth parameters as well as the food and oviposition preference of *T. neocaledonicus* at different rates. Ballhorn et al. (2008) quantified the defense mechanism of lima bean to herbivores and found a substantial variation among 16 lima bean genotypes for cyanide and VOC emissions as defense mechanisms.

Thus, our findings support the hypothesis that lima bean genotypes may affect the performance, populational parameters and the host selection of *T. neocaledonicus*, presenting itself as the first study to describe sources of resistance to pest mites in lima bean in Brazil. Therefore, the results of the present study may be characterized as a basis for further studies aiming to develop a strategy of host plant resistance for IPM programs.

## Author Contributions

SdF and AG-N conceived and designed the research. RG provided the lima bean genotypes. JdSM, PS, MB, SdF, RG, and AG-N wrote the manuscript and analyzed all data. All authors read and approved the manuscript.

## Conflict of Interest Statement

The authors declare that the research was conducted in the absence of any commercial or financial relationships that could be construed as a potential conflict of interest.
